# Crystal structure of the post‐fusion core of the *Human coronavirus 229E* spike protein at 1.86 Å resolution

**DOI:** 10.1107/S2059798318008318

**Published:** 2018-09-10

**Authors:** Lei Yan, Bing Meng, Jiangchao Xiang, Ian A. Wilson, Bei Yang

**Affiliations:** ^1^ Shanghai Institute for Advanced Immunochemical Studies, ShanghaiTech University, 393 Middle Huaxia Road, Shanghai201210, People's Republic of China; ^2^ School of Life Science and Technology, ShanghaiTech University, 393 Middle Huaxia Road, Shanghai201210, People's Republic of China; ^3^ Shanghai Institute of Biochemistry and Cell Biology, Chinese Academy of Sciences, 320 Yueyang Road, Shanghai200031, People's Republic of China; ^4^ University of Chinese Academy of Sciences, 19A Yuquan Road, Beijing100049, People's Republic of China; ^5^ Department of Integrative Structural and Computational Biology, The Scripps Research Institute, 10550 North Torrey Pines Road, BCC206, La Jolla, CA92037, USA

**Keywords:** coronavirus, HCoV‐229E, spike protein, post‐fusion core, X‐ray structure, SARS, MERS

## Abstract

*Human coronavirus 229E* (HCoV‐229E) usually causes mild upper respiratory infections in heathy adults, but may lead to severe complications or mortality in individuals with weakened immune systems. Virus entry of HCoV‐229E is mediated by its spike (S) protein, where the S1 domain facilitates attachment to host cells and the S2 domain is involved in subsequent fusion of the virus and host membranes. During the fusion process, two heptad repeats, HR1 and HR2, in the S2 domain assemble into a six‐helix membrane‐fusion structure termed the fusion core. Here, the complete fusion‐core structure of HCoV‐229E has been determined at 1.86 Å resolution, representing the most complete post‐fusion conformation thus far among published human alphacoronavirus (α‐HCoV) fusion‐core structures. The overall structure of the HCoV‐229E fusion core is similar to those of SARS, MERS and HCoV‐NL63, but the packing of its 3HR1 core differs from those of SARS and MERS in that it contains more noncanonical `x' and `da' layers. Side‐by‐side electrostatic surface comparisons reveal that the electrostatic surface potentials are opposite in α‐HCoVs and β‐HCoVs at certain positions and that the HCoV‐229E surface also appears to be the most hydrophobic among the various HCoVs. In addition to the highly conserved hydrophobic interactions between HR1 and HR2, some polar and electrostatic interactions are also well preserved across different HCoVs. This study adds to the structural profiling of HCoVs to aid in the structure‐based design of pan‐coronavirus small molecules or peptides to inhibit viral fusion.

## 1. Introduction

Coronaviruses (CoVs) are enveloped, positive-sense, single-stranded RNA viruses that generally cause upper respiratory and enteric infections in mammals and birds (Enjuanes *et al.*, 2006; Perlman & Netland, 2009). To date, six different strains of CoVs have been found to infect humans (hereafter referred to as HCoVs), and these include *Severe acute respiratory syndrome-related coronavirus* (SARS), *Middle East respiratory syndrome-related coronavirus* (MERS), *Human coronavirus HKU1* (HCoV-HKU1), *Human coronavirus OC43* (HCoV-OC43), *Human coronavirus 229E* (HCoV-229E) and *Human coronavirus NL63* (HCoV-NL63) (de Wilde *et al.*, 2017; Perlman & Netland, 2009). Of these HCoVs, SARS (Marra *et al.*, 2003; Ksiazek *et al.*, 2003; Peiris, Lai *et al.*, 2003; Rota *et al.*, 2003) and MERS (de Groot *et al.*, 2013; Zaki *et al.*, 2012) are notorious for their high mortality rates [10% for SARS (Peiris, Yuen *et al.*, 2003; Stadler *et al.*, 2003) and 35% for MERS (World Health Organization, 2016)] and pandemic potential. HCoV-229E, on the other hand, is endemic and is frequently associated with respiratory diseases ranging from the common cold to atypical pneumonia (van der Hoek, 2007). In healthy adults, HCoV-229E infection usually causes mild symptoms, including headache, cough, fever and rhinitis (Greenberg, 2016), but can lead to compromised productivity in infected individuals and result in a large impact on the economy. Moreover, HCoV-229E infection can result in severe or even lethal outcomes in infants, young children, seniors and those with weakened immune systems (Forgie & Marrie, 2009; Jartti *et al.*, 2011). Therefore, it is important to investigate the structure and function of HCoV-229E to aid in the development of preventative or therapeutic strategies.

As enveloped viruses, CoVs deliver their viral genomes into the host cytoplasm by fusing the viral membrane with either the host cell or endosome membrane (Belouzard *et al.*, 2012; Hulswit *et al.*, 2016). The spike glycoprotein (S) is an ~500 kDa homotrimer on the envelope surface of CoVs that plays a fundamental role in cell entry, including receptor binding and membrane fusion (Li, 2016). By analogy to other class I viral fusion proteins, such as influenza virus haemagglutinin (Wilson *et al.*, 1981) or the Env protein of HIV-1 (Julien *et al.*, 2013; Lyumkis *et al.*, 2013), the S protein of CoVs can be roughly divided into two structural and functional domains: an N-terminal S1 domain for engagement with cellular receptors and a C-terminal S2 domain for membrane fusion (Belouzard *et al.*, 2012; Li, 2016). The S2 domain contains a fusion peptide (FP), two heptad repeats (HRs) and a transmembrane helix (TM), arranged as FP–HR1–HR2–TM (Liu *et al.*, 2004).

CoV S proteins are synthesized as a single polypeptide chain, and a two-step proteolysis of the S protein prior to viral entry is pivotal for viral–host membrane fusion (Belouzard *et al.*, 2009; Millet & Whittaker, 2014; Park *et al.*, 2016). Specifically, proteolytic cleavage at the S1–S2 domain junction removes the structural constraint of S1 on S2 (Li, 2016; Belouzard *et al.*, 2012; Millet & Whittaker, 2015), a process in which binding of the CoV S1 domain to its cognate receptor may also contribute (Kirchdoerfer *et al.*, 2016; Yuan *et al.*, 2017; Zelus *et al.*, 2003; Matsuyama & Taguchi, 2002). At the same time, cleavage at an additional S2′ site also helps to untether the fusion peptide (FP; Li, 2016; Millet & Whittaker, 2015; Belouzard *et al.*, 2012). Following this two-step cleavage, the otherwise buried hydrophobic FP becomes exposed and available to insert into the host cell membrane (Yao *et al.*, 2016). Subsequent conformational rearrangements within S2 result in the formation of the 3HR1–3HR2 six-helical bundle, thus bringing viral and cellular membranes into close proximity and facilitating membrane fusion (Baquero *et al.*, 2013; Heald-Sargent & Gallagher, 2012).

As illustrated previously, the six-helical bundles from SARS, MERS and HCoV-NL63 feature an extended triple-helical coiled coil (HR1) surrounded by three antiparallel HR2 $\alpha$-helices (Supekar *et al.*, 2004; Xu *et al.*, 2004; Duquerroy *et al.*, 2005; Zheng *et al.*, 2006; Gao *et al.*, 2013; Lu *et al.*, 2014). Such a conformation represents the post-fusion state of CoV spike proteins and is known as the fusion-core structure. More recently, the metastable pre-fusion states of *Mouse hepatitis virus* (MHV), HCoV-HKU1, HCoV-NL63, SARS and MERS have also been determined to high resolution by cryo-EM (Walls, Tortorici, Bosch *et al.*, 2016; Kirchdoerfer *et al.*, 2016; Walls, Tortorici, Frenz *et al.*, 2016; Gui *et al.*, 2017; Yuan *et al.*, 2017). In the pre-fusion state, the S1 domains of CoVs all reside above the trimeric S2 stalk, thereby exerting structural constraints on S2 (Walls, Tortorici, Bosch *et al.*, 2016; Walls, Tortorici, Frenz *et al.*, 2016; Kirchdoerfer *et al.*, 2016; Gui *et al.*, 2017; Yuan *et al.*, 2017). In contrast to their extended helical conformations in the post-fusion state (Xu *et al.*, 2004; Gao *et al.*, 2013; Duquerroy *et al.*, 2005; Supekar *et al.*, 2004; Zheng *et al.*, 2006; Lu *et al.*, 2014), the HR1 motifs within S2 from HCoVs form several shorter helices in their pre-fusion state (Yuan *et al.*, 2017; Walls, Tortorici, Frenz *et al.*, 2016; Gui *et al.*, 2017). Together, these snapshots of the pre-fusion and post-fusion states of S proteins further consolidate the prevailing paradigm on CoV membrane fusion and enhance our knowledge about this complex membrane-fusion apparatus.

Owing to its pivotal role in the host entry of CoVs, the spike protein has been a focus of anti-HCoV drug development. To date, a handful of viral inhibitors that target the spike proteins of SARS and MERS have been reported (Hu *et al.*, 2005; Han *et al.*, 2006; Yi *et al.*, 2004; Bosch *et al.* 2004; Liu *et al.*, 2004; Lu *et al.*, 2014, 2015; Gao *et al.*, 2013; Channappanavar *et al.*, 2015). A subgroup of these inhibitors specifically target the interaction between the S1 domain of the SARS spike protein and its corresponding host receptors (Hu *et al.*, 2005; Han *et al.*, 2006). The remainder of these inhibitors target HR1s in the S2 domain of SARS and MERS by mimicking the corresponding HR2s, thereby inhibiting formation of the 3HR1–3HR2 helical bundle (Bosch *et al.*, 2004; Liu *et al.*, 2004; Lu *et al.*, 2014, 2015; Gao *et al.*, 2013; Channappanavar *et al.*, 2015). Both groups of inhibitors are effective against specific HCoVs, at least *in vitro*. Nevertheless, the hypervariable nature of the S1 domain and the highly conserved features of the S2 domain render the latter a more suitable target for the development of pan-CoV inhibitors. As CoVs are rapidly evolving (Forni *et al.*, 2016), pandemic outbreaks of newly emerging HCoVs become possible when zoonotic CoVs are able to accumulate sufficient mutations to cross the species barrier (Ge *et al.*, 2013; Menachery *et al.*, 2015, 2016). Thus, a broad-spectrum anti-HCoV drug or vaccine would be more effective than a specific one. To this end, structural profiling of the evolutionarily conserved S2 domain, especially the 3HR1–3HR2 six-helical bundle that drives the membrane-fusion process, would undoubtedly provide valuable information to help in the design of such a broad-spectrum anti-HCoV HR2 mimic.

Of the six HCoVs, HCoV-NL63 and HCoV-229E belong to the alphacoronavirus ($\alpha$-CoV) family, while the other four HCoVs are classified as betacoronaviruses ($\beta$-CoVs) (Woo *et al.*, 2009). Among them, HCoV-229E was the first to be identified (Hamre & Procknow, 1966). However, its spike protein has been the least structurally studied. The host receptor of HCoV-229E was revealed to be human aminopeptidase N (APN) more than two decades ago (Yeager *et al.*, 1992). Nevertheless, structural elucidation of the interaction between HCoV-229E and APN was only revealed very recently (Wong *et al.*, 2017). More recently, a crystal structure of the HCoV-229E fusion core (HR1 residues 788–838 and HR2 residues 1060–1097) was reported at 2.45 Å resolution (Zhang *et al.*, 2018) while we were preparing this manuscript. Here, we present a more complete fusion-core structure of HCoV-229E at 1.86 Å resolution (HR1 residues 785–873 and HR2 residues 1052–1105) with an eight-turn longer HR1 helix and at higher resolution. The fusion-core structure of HCoV-229E is similar overall to those of SARS, MERS and HCoV-NL63. Nevertheless, since HCoV-229E has a longer HR1 and HR2, the packing of its hydrophobic core and the buried interface between HR1 and HR2 differ from those in SARS and MERS (Supekar *et al.*, 2004; Lu *et al.*, 2014; Xu *et al.*, 2004; Duquerroy *et al.*, 2005; Gao *et al.*, 2013) and are more similar to those in HCoV-NL63, another human $\alpha$-CoV (Zheng *et al.*, 2006). Structural studies of the HCoV spike protein may be used to develop effective vaccines and drugs against HCoVs. We thus anticipate that our structure and the recently solved fusion-core structure of HCoV-229E, along with those of SARS, MERS and NL63, may serve as a template for the design of structure-based pan-coronavirus small molecules or peptides to inhibit viral fusion.

## 2. Materials and methods

### 2.1. Plasmid construction

To express the HCoV-229E fusion core, the coding sequences for HR1 (residues 785–873) and HR2 (residues 1052–1105) were connected *via* a short linker (encoding L6; SGGRGG) by overlapping PCR. The resulting sequence HR1-L6-HR2 was then subcloned into the pET-28a vector with an N-terminal in-frame SUMO tag. The final construct thus contains an N-terminal $His_6$ tag followed by the SUMO domain including the recognition sequence for Ulp1 and the coding sequence for HR1-L6-HR2.

### 2.2. Protein production and purification

For protein production, the pET-28-SUMO-HR1-L6-HR2 plasmid was transformed into *Escherichia coli* strain BL21 (DE3) competent cells. A single colony was inoculated into 5 ml Luria–Bertani (LB) medium containing 100 µg ml$^{-1}$ kanamycin (AMRESCO) and incubated overnight at 37°C. The overnight culture was then seeded into 2 l fresh LB medium and cultured at 37°C until the $OD_{600}$ (optical density at 600 nm) reached 0.6. Target protein overexpression was then induced with 1 m*M* isopropyl $\beta$-D-1-thiogalactopyranoside (IPTG; AMRESCO) at 16°C for 16 h. After harvesting *via* centrifugation at 16 000 rev min$^{-1}$ for 30 min, the cell debris was resuspended in lysis buffer (50 m*M* Tris–HCl pH 7.5, 300 m*M* NaCl) supplemented with phenylmethylsulfonyl fluoride (PMSF; Biovision) and lysed using a sonicator (Thermo Fisher Scientific). The cell lysate was then centrifuged at 70 000*g* for 20 min at 4°C. The supernatant was collected and loaded onto TALON metal-affinity resin (Clontech). After extensive washing, the HR1-L6-HR2 protein of interest was eluted with lysis buffer supplemented with 500 m*M* imidazole. The fractions eluted from the TALON column were then dialyzed against dialysis buffer (20 m*M* Tris–HCl pH 8.0, 5 m*M* $\beta$-mercaptoethanol, 150 m*M* NaCl) overnight at 4°C and then processed with Ulp1 [at a 1:100(*w*:*w*) ratio] to remove the SUMO tag from HR1-L6-HR2. Finally, the cleaved product was applied onto a Superdex 75 gel-filtration column (GE Healthcare). Fractions containing a homogeneous HR1-L6-HR2 trimer were collected and concentrated by ultrafiltration using Amicon Ultra-4 10 kDa centrifugal filter units (Millipore). All chemicals were from Sigma–Aldrich unless otherwise specified.

### 2.3. Crystallization, data collection and structure determination

The HR1-L6-HR2 fusion protein was crystallized at 20°C using the hanging-drop vapour-diffusion method. Crystals were grown on a siliconized cover slip by equilibrating a mixture consisting of 1 µl protein solution (8 mg ml$^{-1}$ HR1-L6-HR2 trimer in 20 m*M* Tris–HCl pH 8.0, 150 m*M* NaCl) and 1 µl reservoir solution (0.03 *M* citric acid, 0.07 *M* bis-tris propane pH 7.6, 20% PEG 3350) against 400 µl reservoir solution. Single crystals grew after one week and were flash-cooled in liquid nitrogen for data collection after adding 20% glycerol as a cryoprotectant.

Diffraction data were collected on beamline BL17U1 of the Shanghai Synchrotron Radiation Facility (SSRF) using a wavelength of 0.9795 Å and an ADSC Q315r CCD detector. 360 images were recorded at a distance of 250 mm with a 1° oscillation angle and an exposure time of 1.0 s. Raw data were indexed and processed using *HKL*-3000 in space group *R*32:*H*. Molecular replacement was performed with *Phaser* in *PHENIX* (Adams *et al.*, 2010) using the HCoV-NL63 fusion-core structure (PDB entry 2ieq; Zheng *et al.*, 2006) as a search model. The initial model was further improved by cycles of manual building and refinement using *Coot* (Emsley *et al.*, 2010) and *PHENIX* (Adams *et al.*, 2010). Torsion-angle NCS restraints and TLS groups were used during refinement. The quality of the final model was analysed with *MolProbity* (Chen *et al.*, 2010). Data-collection and refinement statistics are shown in Table 1. Atomic coordinates and structure factors have been deposited in the Protein Data Bank (PDB entry 5yl9). Figures were prepared with *PyMOL* (http://www.pymol.org). Electrostatic calculations were performed with *PDB2PQR* (Dolinsky *et al.*, 2004). The volumes of cavities and pockets were calculated with the *CASTp* 3.0 online server (Dundas *et al.*, 2006). Pitch values were calculated with *TWISTER* (Strelkov & Burkhard, 2002).

## 3. Results

### 3.1. Overall architecture of the HCoV-229E fusion core

The HR1 (residues 785–873) and HR2 (residues 1052–1105) regions of HCoV-229E were connected *via* a six-amino-acid linker (L6; SGGRGG), thereby generating an HR1-L6-HR2 fusion protein (Fig. 1*a*). Similar linkers have been utilized in

**Table 1**
X-ray data-collection and refinement statistics.

Values in parentheses are for the outer shell.

| | |
|---|---|
| Space group | $R32{:}H$ |
| $a$, $b$, $c$ (Å) | 46.9, 46.9, 402.1 |
| $\alpha$, $\beta$, $\gamma$ (°) | 90, 90, 120 |
| Wavelength (Å) | 0.9795 |
| Resolution range (Å) | 44.7–1.86 (1.93–1.86) |
| No. of unique reflections | 15101 (1465) |
| Completeness (%) | 99.9 (100) |
| Multiplicity | 20.9 (21.6) |
| $\langle I/\sigma(I)\rangle$ | 31.5 (5.3) |
| $R_{meas}$† (%) | 7.5 (67.0) |
| $R_{p.i.m.}$‡ (%) | 1.6 (14.3) |
| $CC_{1/2}$§ | 0.964 |
| Overall Wilson $B$ value (Å$^2$) | 24 |
| No. of reflections (work) | 14231 (1379) |
| No. of reflections (test) | 764 (86) |
| $R_{cryst}$¶ (%) | 18.4 (22.0) |
| $R_{free}$¶ (%) | 20.6 (25.5) |
| No. of non-H atoms | |
| Protein | 1117 |
| Water | 115 |
| R.m.s. deviations | |
| Bonds (Å) | 0.008 |
| Angles (°) | 1.00 |
| Average $B$ values (Å$^2$) | |
| Protein | 30 |
| Water | 43 |
| Ramachandran plot | |
| Most favoured (%) | 99.29 |
| Allowed (%) | 0.71 |

† $R_{meas} = \sum_{hkl}\{N(hkl)/[N(hkl)-1]\}^{1/2}\sum_i |I_i(hkl) - \langle I(hkl)\rangle| / \sum_{hkl}\sum_i I_i(hkl)$. ‡ $R_{p.i.m.} = \sum_{hkl}\{1/[N(hkl)-1]\}^{1/2}\sum_i |I_i(hkl) - \langle I(hkl)\rangle| / \sum_{hkl}\sum_i I_i(hkl)$. § $CC_{1/2}$ is the Pearson correlation coefficient between two random half data sets for the outer shell. ¶ $R_{meas} = \sum_{hkl}\big||F_{obs}| - |F_{calc}|\big| / \sum_{hkl}|F_{obs}|$; $R_{free}$ is defined as $R_{cryst}$ calculated from 5% of reflections that were excluded during refinement.

structural studies of MHV and HCoV-NL63 fusion cores and were found not to affect the intrinsic interaction (or packing) between HR1 and HR2 (Xu *et al.*, 2004; Zheng *et al.*, 2006). The HR1-L6-HR2 construct crystallized in space group $R32{:}H$, with unit-cell parameters $a = b = 46.9$, $c = 402.1$ Å and one molecule per asymmetric unit (Table 1). The structure was solved by molecular replacement using the crystal structure of the HCoV-NL63 fusion core (PDB entry 2ieq) as the search model, and was refined to a final resolution of 1.86 Å with an $R_{cryst}$ of 18.4% and an $R_{free}$ of 20.6% (Table 1).

HR1 of HCoV-229E forms a 24-turn $\alpha$-helix, while HR2 of HCoV-229E adopts a mixed conformation: residues 1067–1098 fold into a nine-turn $\alpha$-helix, while residues 1052–1066 and 1099–1105 on either side of the helix adopt an extended conformation (Fig. 1*b*). The HR1 regions of three HR1-L6-HR2 molecules are arranged around the crystallographic threefold symmetry axis and form the central hydrophobic core (Fig. 1*c*). The three companion HR2 regions pack on the outside into the hydrophobic grooves that are formed between adjacent HR1 helices (Figs. 1*c* and 1*d*) in an antiparallel manner, resulting in a six-helical bundle structure with dimensions of ~128 Å in length and 32 Å in diameter with a left-handed supercoil (Fig. 1*c*). Such a structure is typical for the CoV fusion core and represents the post-fusion state of the HCoV-229E spike protein. As our structure is at higher resolution and the HR1 and HR2 motifs are more complete than in Zhang *et al.* (2018), we therefore used our HCoV-229E structure in the following comparisons of different HCoVs.

### 3.2. Hydrophobic core packing of the long HR1 from HCoV-229E

Sequence alignment of HCoVs indicates that a 14-amino-acid insertion arises in the HR1 regions of HCoV-NL63 and HCoV-229E (Fig. 1*f*) that would make the HR1 helix of HCoV-229E four turns (two heptad repeats) longer than those of MERS and SARS (Fig. 1*e*; insertion highlighted in red). To compensate for their longer HR1 regions, the HR2 region of HCoV-229E or HCoV-NL63 also contains a 14-residue insertion (Fig. 1*g*) that contributes to a longer HR2 helix (Fig. 1*e*; insertion highlighted in yellow).

The 24-turn $\alpha$-helix of HCoV-229E HR1 is the longest among all HCoV fusion-core structures published thus far (Fig. 2*a*). 26 hydrophobic residues as well as three polar residues (Gln791, Thr819 and Gln840) from each HR1 line the coiled-coil interface and pack in layers to form the hydrophobic 3HR1 core of HCoV-229E (Fig. 2*a*, first panel). Besides the canonical 'a' and 'd' layers, which are a feature of heptad repeats, Zheng and coworkers introduced the concept of 'x' and 'da' (or 'y') layers to illustrate the noncanonical packing modes observed in the structure of the HCoV-NL63 fusion core (Zheng *et al.*, 2006). Such 'x' and 'da' packing modes are also observed here for the 3HR1 core of HCoV-229E (Fig. 2*a*, first panel). Interestingly, the four additional turns that are unique to $\alpha$-HCoVs (Fig. 1*f*) all adopt noncanonical packing geometry, with 'd/a' layers alternating with 'x' layers (Fig. 2*a*, highlighted in red). Re-analysis of published SARS and MERS fusion cores reveals that 'x' and 'da' mode packing also exists in the 3HR1 cores of MER and SARS, albeit less prevalently than in $\alpha$-HCoVs (HCoV-229E and HCoV-NL63) (Fig. 2*a*). In the standard heptad repeats, although the 'a' or 'd' residues (knobs) interact around the trimer threefold axis, they do so in such a way that their side chains are more laterally arranged and fit in between the 'a' and 'g' residues (holes) or the 'd' and 'e' residues (holes), respectively (Fig. 2*b*, panels 1 and 2). In the noncanonical 'x' layer, each packing residue points its side chain towards the centre of the threefold symmetry axis (Fig. 2*b*, third panel); while in the 'da' layer the 'a' and 'd' packing residues are arranged in a pseudo-hexagonal manner through hydrophobic interactions, thereby leaving a large unfilled space in the middle (Fig. 2*b*, fourth panel). As a consequence, a central cavity is always seen in the 'da' layer (Fig. 2*a*; cavity shown as a yellow surface). As calculated with the *CASTp* 3.0 server (Dundas *et al.*, 2006), the molecular surface volume distribution of these cavities varies in different HCoVs. For HCoV-229E, the largest cavity seen in the Ala815/Ile816 'da' layer has a volume of 228 Å$^3$, while the volumes of the cavities in the other 'da' layers all fall between 20 and 50 Å$^3$; for HCoV-NL63, the largest cavity was in the Ala996/Ile997 'da' layer, with a volume of 206 Å$^3$, and the other cavities in 'da' layers are all smaller than 55 Å$^3$; for MERS, the largest cavity in the Asn1029/Ala1030 'd/a' layer has a volume of 266 Å$^3$ and no other cavity larger than 50 Å$^3$

was present in the structure. In sharp contrast to HCoV-229E, HCoV-NL63 and MERS, the cavities seen in the three 'da' layers of the SARS 3HR1 core are all large, with volumes of 105, 232 and 285 Å$^3$, respectively.

**Figure 1**
Overall structure of the HCoV-229E fusion core. (*a*) Schematic diagram illustrating the design of the HR1-L6-HR2 fusion-core construct. (*b*, *c*) Monomeric (*b*) and trimeric (*c*) structures of the HR1-L6-HR2 fusion protein. The red pole (left panel) and filled triangle (right panel) in (*c*) represent the trimer threefold axis. (*d*) The packing of HR2 against the central hydrophobic core of HR1 as illustrated by a solvent-accessible surface rendering. The solvent-accessible surface is coloured according to the electrostatic potential, which ranges from +5 V (most positive, dark blue) to −5 V (most negative, dark red), with hydrophobic in white. (*e*) The HR2 helices are depicted as dark grey ribbons on the light grey surface of the HCoV-229E 3HR1 core to highlight the corresponding insertions in HR1 and HR2 in red and yellow, respectively. (*f*, *g*) Sequence alignment of the HR1 region (*f*) and HR2 region (*g*) of HCoV-229E, HCoV-NL63, HCoV-HKU1, HCoV-OC43, SARS and MERS. The HR1 and HR2 coverages of previous HCoV structures (PDB entry 2ieq for HCoV-NL63, PDB entry 1wyy for SARS and PDB entry 4njl for MERS) and the recently reported HCoV-229E structure (PDB entry 5zhy; Zhang *et al.*, 2018) are depicted with a yellow background. The HCoV-229E structure presented here is denoted with a cyan highlight. The filled squares and stars underneath the residues highlight the conservation of residues in HR2 that are involved in hydrophobic interactions between HR1 and HR2 in the various HCoVs.

The presence of multiple large cavities (>100 Å$^3$) in the middle of the 3HR1 core of SARS may suggest that the supercoils of SARS are less stable compared with the supercoils of HCoV-229E and HCoV-NL63. We thus calculated the average pitch values of different HCoVs using *TWISTER* (Strelkov & Burkhard, 2002). The pitch values of the HCoV-229E, HCoV-NL63 and MERS supercoils are 188.0, 189.3 and 187.2 Å, respectively, which are all similar to each other and close to the most commonly seen pitch of trimeric coiled-coil structures (Seo & Cohen, 1993). Nevertheless, the 194.7 Å pitch value of the SARS supercoil is longer than those of MERS, HCoV-229E and HCoV-NL63, indicating that the 3HR1 core of SARS is less twisted compared with the others.

Side-by-side electrostatic surface comparisons also reveal substantial differences between α-HCoVs and β-HCoVs (Fig. 2*c*). The electrostatic surface of SARS and MERS are strikingly similar to each other, except for some differences in the lower region (boxed with black dashed lines in Fig. 2*c*). The electrostatic surfaces

**Figure 2**
Differences in the 3HR1 core between α-HCoVs and β-HCoVs. (*a*) A side-by-side comparison illustrates the differences in the heptad-repeat packing geometry of HCoV-229E, HCoV-NL63 (PDB entry 2ieq), SARS (PDB entry 1wyy) and MERS (PDB entry 4njl). The hydrophobic 3HR1 core is depicted as a grey ribbon, cavities as a yellow surface mesh and the side chains of residues at 'd/a' positions as cyan stick models. The helical turns are numbered from the N-terminus to the C-terminus on one of the HR1 helices in the trimer. On the left of each ribbon representation, the packing residues lining the trimeric coiled-coil interface are indicated in black, with residues at the 'a' and 'd' positions (canonical 'a' and 'd' layer) boxed in beige, residues at 'x' positions ('x' layer) in magenta and residues at 'd/a' positions ('da' layer) in cyan. (*b*) From left to right: cross-sections of representative 'a', 'd', 'x' and 'da' layers in the 3HR1 core of HCoV-229E. The $2F_o - F_c$ electron-density maps are contoured at 2σ. (*c*) Electrostatic surfaces illustrating differences in the 3HR1 core from HCoV-229E, HCoV-NL63, SARS and MERS. The red dashed boxes depict the regions on different 3HR1 cores where the electrostatic surface potentials are opposite in α-HCoVs and β-HCoVs. The black dashed boxes highlight the only regions on SARS and MERS where the electrostatic surface potentials are different.

of HCoV-229E and HCoV-NL63 are also alike to some extent, but the 3HR1 core of HCoV-229E is more hydrophobic. In fact, the 3HR1 core of HCoV-229E seems to be the most hydrophobic among the four HCoVs. It is worth noting that at certain positions the electrostatic surface potentials are opposite in $\alpha$-HCoVs and $\beta$-HCoVs (Fig. 2*c*; boxed with red dashed lines). Considering that both hydrophobic and electrostatic interactions between HR1 and HR2 contribute to the affinity between them (Aydin *et al.*, 2014), such differences in electrostatic surface potentials should be taken into consideration for the design and development of a pan-CoV inhibitory HR2 mimic.

### 3.3. Interaction between HR1 and HR2 of HCoV-229E

The HR2 residues 1067–1097 of HCoV-229E fold into a nine-turn amphipathic $\alpha$-helix (Fig. 1*b*), which fits snugly onto the 3HR1 core mainly through extensive hydrophobic interactions (Fig. 3*a*). Specifically, residues at the 'd' position (Leu1067, Leu1074, Leu1081, Leu1088 and Ile1095) in this nine-turn $\alpha$-helix bury their side chains (Fig. 3*a*; shown as orange stick models) into hydrophobic pockets on the surface of the 3HR1 core of HCoV-229E. Another kind of hydrophobic packing was also observed at locations where the hydrophobic surface on the 3HR1 core is relatively flat (Fig. 3*a*; hereafter termed ridges). At these locations, residues at the 'a' (Ile1071, Ser1078, Val1085, Ile1092 and Leu1099) and 'g' (Glu1070, Lys1077, Thr1084, Leu1091 and Thr1098) positions on the HR2 helix pack about 50% of the solvent-accessible surface area (SASA) of their side chains (Fig. 3*a*; shown as yellow stick models) against the HR1 residues constituting these ridges. Besides the nine-turn $\alpha$-helix, the rest of HR2 adopts an extended conformation which also makes substantial hydrophobic interactions with the 3HR1 core. Interestingly, only the first type of hydrophobic interaction was seen in these regions (Fig. 3*a*), where the side chains of Pro1053, Leu1055, Val1057, Tyr1060, Leu1065, Val1100, Leu1102 and Trp1104 (orange stick models in Fig. 3*a*) are completely buried in the cavities on the 3HR1 core. Overall, the side chains of hydrophobic residues from the HR2 helix of HCoV-229E fit tightly into the cavities and ridges on the 3HR1 core, almost like chains onto a sprocket (Fig. 3*b*). It is worth noting that the HR2 residues that either fill the hydrophobic cavities or mask the hydrophobic ridges on the 3HR1 core are largely conserved across the different HCoVs (Fig. 1*g*; denoted by stars and filled squares, respectively), corroborating the essential role played by hydrophobic interactions in the interaction between HR1 and HR2.

Besides hydrophobic packing, electrostatic and polar interactions also contribute to the affinity and specificity between HR1 and HR2 (Fig. 4). In the helical region, residues

**Figure 3**
Two types of hydrophobic interactions are observed between the HR1 and HR2 helices. (*a*) Left panel: electrostatic surface illustrating the hydrophobic cavities on the surface of the HCoV-229E 3HR1 core. The lower half of the 3HR1 core is rotated for better display of the hydrophobic cavities in this region. Right panel: HR2 helices are shown as teal ribbons on the dark grey surface of the HCoV-229E 3HR1 core. HR2 residues that bury their side chains completely into the cavities on HR1 are shown as orange stick models and HR2 residues that pack around 50% of the solvent-accessible surface of their side chains on ridges of HR1 are depicted as yellow stick models. (*b*) Surface representation of HR1 and HR2 helices illustrating that HR2 residues fit snugly onto the surface of the 3HR1 core, thereby filling hydrophobic cavities in HR1 and masking its hydrophobic surface. The 3HR1 core is shown as an electrostatic surface, and HR2 residues involved in hydrophobic interactions are depicted as orange (completely buried) and yellow (packing, ~50% buried) surfaces, respectively.

**Figure 4**

Comparison of different CoV fusion cores reveals differences and similarities in the interactions between their HR1 and HR2 motifs. Cartoon representations illustrating the side-chain electrostatic and polar interactions (*a*) and main-chain polar interactions (*b*, *c*) between HR1 and HR2 in HCoV-229E, HCoV-NL63 (PDB entry 2ieq), SARS (PDB entry 1wyy) and MERS (PDB entry 4njl). Interactions between HR1 residues and the main-chain atoms of HR2 are only seen in the N-terminal (*c*) and C-terminal (*b*) extended regions of HR2. The HR1 motifs are depicted as white ribbons and the HR2 motifs as green ribbons. For clarity, only two HR1 motifs of the 3HR1 core are shown. The HR1 and the HR2 residues that are involved in interactions are illustrated as stick models, with black dashed lines representing hydrogen bonds and/or salt bridges. The residues are colour-coded as follows: HR1, whitish grey; HR2, teal (HCoV-229E), hot pink (HCoV-NL63), gold (MERS) and slate (SARS). Interactions that are conserved across different HCoVs are shown as yellow stick models. All polar interactions of HCoV-229E are clearly labelled, whereas only conserved interactions are labelled for the other HCoVs. The labels of HR1 residues are differentiated from those of HR2 residues by a prime after the residue number.

on HR2 mainly engage residues on HR1 through side-chain to side-chain interactions that include hydrogen bonds and salt bridges (Fig. 4*a*). In the extended region of HR2, extensive polar interactions were observed between the main-chain atoms on HR2 and the side chains of residues from HR1 (Figs. 4*b* and 4*c*). It is worth noting that some of these interactions are highly conserved across different HCoVs (Fig. 4; conserved interactions are shown as yellow stick models, with black dashed lines indicating hydrogen bonds or salt bridges). Interestingly, these conserved interactions are mainly located in the regions adjacent to the end of the nine-turn helix, suggesting that they might play a critical role in fixing the register of the HR2 helical region on the HR1 surface.

## 4. Discussion

We have determined the fusion-core structure of HCoV-229E at 1.86 Å resolution, thus further completing the structural profiles of HCoV fusion cores. The HCoV-229E structure reveals that its HR1 folds into an unusually long 24-turn helix, which is the longest observed so far in published HCoV fusion-core structures (Fig. 1). Currently, multiple fusion-core structures are available for SARS and MERS and each has a different HR1/HR2 coverage (or completeness) (Supekar *et al.*, 2004; Lu *et al.*, 2014; Xu *et al.*, 2004; Duquerroy *et al.*, 2005; Gao *et al.*, 2013). Among them, the most complete post-fusion structures are for SARS (PDB entry 1wyy; Duquerroy *et al.*, 2005) and MERS (PDB entry 4njl; Lu *et al.*, 2014), with a 21-turn and 22-turn HR1 helix, respectively (Figs. 1*f* and 2*a*). Both of these HR1s are shorter than the 24-turn HR1 reported here, as the HR1 helices of $\alpha$-HCoVs are intrinsically longer than those of $\beta$-HCoVs owing to a 14-residue insertion in their HR1 domains (Fig. 1*f*). The 14-turn and 13-turn HR1 helices seen in previous post-fusion structures of HCoV-NL63 and HCoV-229E cover only around 56% of their annotated HR1 sequences (Fig. 1*f*; coverage highlighted with a yellow background; amino acids 981–1031 *versus* 964–1054 for HCoV-NL63 and amino acids 788–838 *versus* 783–873 for HCoV-229E; Zheng *et al.*, 2006; Zhang *et al.*, 2018). In contrast, for the structure presented here the HR1 coverage is 97% (Fig. 1*f*; coverage highlighted with a cyan background; amino acids 785–872 *versus* 783–873), which represents the most complete post-fusion conformation among all published $\alpha$-HCoV fusion-core structures (Figs. 1*f* and 1*g*).

Owing to the 14-residue insertion in the HR1 regions, the HR2 regions of $\alpha$-HCoVs are also intrinsically longer than those of $\beta$-HCoVs. The HR2 helix of HCoV-229E consists of nine turns (Fig. 4), whereas in $\beta$-HCoVs such as MERS and SARS the HR2 helix is usually only 4–5 turns (Fig. 4). As a functional outcome, the melting temperatures ($T_{\rm m}$) of $\alpha$-HCoVs are significantly and consistently higher than those of $\beta$-HCoVs, presumably owing to a larger buried interface and a higher affinity between the longer HR2s and 3HR1 cores of $\alpha$-HCoVs. In detail, the $T_{\rm m}$ values for the HCoV-229E and HCoV-NL63 post-fusion cores are 90.1 and 98.0°C, respectively (Xia *et al.*, 2018; Zheng *et al.*, 2006), while the $T_{\rm m}$ values for SARS and MERS are 85.0 and 87.0°C, respectively (Xu *et al.*, 2004; Lu *et al.*, 2014).

The fusion-core structure of HCoV-229E and those of SARS, MERS and HCoV-NL63 are similar to each other overall (Fig. 2*a*), despite differences in the packing of their 3HR1 cores. Specifically, $\alpha$-HCoVs seem to have more non-canonical 'x' and 'da' layers than $\beta$-HCoVs, especially in the region of the 14-residue insertion (Figs. 2*a* and 2*b*). Compared with the 3HR1 cores of MERS, HCoV-NL63 and HCoV-229E, the SARS supercoils are less twisted, as shown by the longer pitch and larger hydrophobic voids in the middle of the 3HR1 core. Moreover, side-by-side electrostatic surface comparisons of the 3HR1 cores also reveal substantial differences between $\alpha$-HCoVs and $\beta$-HCoVs (Fig. 2*c*). Notably, at certain positions, the electrostatic surface potentials are opposite in $\alpha$-HCoVs and $\beta$-HCoVs (Fig. 2*c*).

Similar to other HCoVs, the high-affinity interaction between HR1 and HR2 of HCoV-229E is mediated by both extensive hydrophobic and hydrophilic interactions (Figs. 3 and 4). Besides the highly conserved hydrophobic interactions, some polar and electrostatic interactions are also well preserved across different HCoVs (Fig. 4). Together, these conserved interactions and the differences in the electrostatic surface potential of 3HR1 cores across different HCoVs should be taken into consideration when designing pan-CoV inhibitors that mimic HR2 for targeting HR1. Although previous studies have found that peptides derived from the HR2s of SARS or MERS can prevent the host entry of the corresponding viruses, these peptides lack cross-inhibitory activity with each other or with other HCoVs (Liu *et al.*, 2004; Lu *et al.*, 2014). Since most of the hydrophobic interactions between HR1 and HR2 are highly conserved across all HCoVs, this implies that hydrophilic interactions are also important for the adhesion and register of HR2s onto the 3HR1 cores. Indeed, it has been reported that both hydrophobic and hydrophilic interactions between HR1 and HR2 of SARS contribute to the affinity between them (Aydin *et al.*, 2014). Hence, a good pan-CoV inhibitory peptide mimic of HR2 would preserve all of the conserved hydrophobic and hydrophilic interactions between HR1 and HR2 of all HCoVs and also accommodate the variation among different HCoVs. More specifically, at the positions where the electrostatic surface potentials of 3HR1 cores are opposite in $\alpha$-HCoVs and $\beta$-HCoVs, residues on the HR2 inhibitory peptide mimic should be carefully chosen such that they could form contacts, but not electrostatic or steric clashes, with surrounding residues on the 3HR1 cores of different HCoVs.

The deadly global outbreaks of SARS in 2002 and MERS in 2012 highlight the importance of developing vaccines or treatments for CoVs, for which there are still no effective drugs or vaccines available. Both SARS and MERS are zoonotic CoVs. Before inter-species jumping, humans rarely came into contact with zoonotic CoVs, and therefore lack neutralizing antibodies to zoonotic CoVs. As a consequence, zoonotic CoVs usually pose substantial health and pandemic threats to humans. Given the high genomic mutation rate of CoVs and ever-increasing global population shifts, future outbreaks of CoV pandemics such as SARS or MERS are very likely to occur. To this end, structural and functional characterization of HCoVs takes on greater significance, especially for the structure-based design of future therapies or vaccines.

### Acknowledgements

We thank the staff of the BL17U1 beamline at the Shanghai Synchrotron Radiation Facility for assistance during data collection.

### Funding information

The following funding is acknowledged: National Natural Science Foundation of China (grant No. 31600619 to Bei Yang); Science and Technology Commission of Shanghai Municipality (award No. 16PJ1407500 to Bei Yang).

## Graphical abstract

The complete post‐fusion core structure of the *Human coronavirus 229E* spike protein was determined at 1.86 Å resolution. Comparison of the interactions between heptad repeats HR1 and HR2 in different human coronaviruses reveals some differences, which should be taken into consideration when designing pan‐coronavirus HR2‐mimicking inhibitors that target HR1.

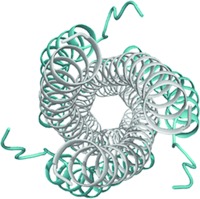

The full text for this article, hosted at http://journals.iucr.org, is unavailable due to technical difficulties.

## Supporting information

Supporting information for this article can be found http://scripts.iucr.org/cgi-bin/paper?ud5004

